# The Spillover of African Swine Fever in Western Poland Revealed Its Estimated Origin on the Basis of O174L, K145R, MGF 505-5R and IGR I73R/I329L Genomic Sequences

**DOI:** 10.3390/v12101094

**Published:** 2020-09-27

**Authors:** Natalia Mazur-Panasiuk, Marek Walczak, Małgorzata Juszkiewicz, Grzegorz Woźniakowski

**Affiliations:** Department of Swine Diseases, National Veterinary Research Institute, Partyzantów 57 Avenue, 24-100 Puławy, Poland; marek.walczak@piwet.pulawy.pl (M.W.); malgorzata.juszkiewicz@piwet.pulawy.pl (M.J.); grzegorz.wozniakowski@piwet.pulawy.pl (G.W.)

**Keywords:** African swine fever virus, molecular epidemiology, next-generation sequencing, western Poland, virus excursion

## Abstract

The African swine fever epidemic occurred in Poland at the beginning of 2014 and, up to date, the disease has been spreading mainly in the eastern part of the country. Unexpectedly, in November 2019 an infected wild boar case was confirmed in Lubuskie voivodship in western Poland. During the following weeks, several dozen African swine fever virus (ASFV)-positive animals were notified in the neighboring area, causing severe concern regarding further spread of the disease to the mostly pig-dense region in Poland, namely, Wielkopolskie voivodship. Moreover, almost a year after, several infected wild boar cases were confirmed for the first time in Germany, just beyond the Polish border, sending out a shock wave through the global pig market. The whole genome sequence of ASFV, isolated from the first case of ASF in western Poland, and three selected viruses from other affected areas, revealed the tandem repeat and single nucleotide polymorphism (SNP) variations in reference to the Georgia 2007/1 strain. These data, supported by the conventional sequencing of selected genomic regions from a total of 154 virus samples isolated between 2017 and 2020 in Poland, shed a new light on pathogen epidemiology. The sequence variations within the *O174L* gene detected in this study showed that cases identified in western Poland might be originating from the so-called southern Warsaw cluster. Moreover, the viruses originating from the northern Warsaw cluster do not possess single nucleotide polymorphism (SNP) mutations within the *K145R* and *MGF 505-5R* genes, which are specific to all of the other Polish ASFV strains. These results led to a conclusion of their distinct origin. Supporting these results, the nucleotide sequencing of *I73R/I329L* intergenic region revealed its new, previously undescribed variant, called IGR IV, with an additional three tandem repeats of 10 nucleotides in comparison to the reference sequence of the Georgia 2007/1 strain.

## 1. Introduction

African swine fever (ASF) is considered as one of the most devastating diseases of pigs and wild boar. During the last years, its rapid spread in Europe and Asia led to severe and difficult-to-estimate economic loss around the world. The virus was introduced to Poland at the beginning of 2014, and since then it has been spreading consequently in the wild boar population, with seasonal spillovers to the domestic pig population [1]. Nevertheless, until the end of 2019 the disease had been reported only among the eastern voivodships of Poland, i.e., Podlaskie, Lubelskie, Mazowieckie, Warmińsko-Mazurskie, Podkarpackie and Świętokrzyskie [1]. On November 6th of 2019, a wild boar killed in a car accident was found in Sława, Wschowa county, Lubuskie voivodship in western Poland. The humeral bone of this animal was collected and sent to the National ASF Reference Laboratory in order to exclude African swine fever virus (ASFV) presence. Unfortunately, the real-time PCR result of the DNA extracted from the bone marrow of this animal confirmed the presence of ASFV genome. This unexpected finding caused rapid and intensified passive and active surveillance actions in neighboring counties, resulting in a total of 130 ASFV infected animals confirmed up to the end of 2019 within this area, including eight counties in three voivodeships (namely: Lubuskie, Dolnośląskie and Wielkopolskie). During the subsequent months, the disease spread among other areas of western Poland. In the first half of 2020, in total 1683 ASFV-positive wild boars were confirmed there. Consequently, the incidence of ASF in wild boar took a toll of almost 34,000 domestic pigs which were stamped out due to the ASF outbreaks at six individual farms (data of 27.08.2020) [1]. Almost a year later, in September 2020, the disease crossed the German border, which electrified the worldwide pig market and caused severe concern about its further westward spread.

Several next- as well third- generation sequencing platforms, including Roche 454, Illumina, IonTorrent, Pacbio and MinIon technologies have repeatedly been applied to generate whole genome sequences of numerous ASFV strains [2,3,4,5,6,7,8]. In order to determine the geographical origin of new ASFV cluster in western Poland, which was distanced about 270 km westwards from the nearest confirmed cases, next generation sequencing (NGS) of four selected ASFV isolates collected between 2017–2019 in Poland, including samples originated from western cluster was performed. Based on the obtained result, supported by other investigations [2,6,9,10], the *O174L*, *K145R*, *MGF 505-5R* genes and intergenic region (IGR) *I73R/I329L* region were selected as molecular “fingerprints”. The conducted conventional sequencing of these ASFV genomic regions allowed for the enhanced discrimination between closely related viruses circulating within the country since 2014. The obtained results provide in-depth insight into the ASF epidemiology in Poland.

The alignment of sequences covering the intergenic region between *I73R* and *I329L* genes in ASFV isolates from eastern and Central Europe revealed the presence of a 10 nt insertion of a tandem-repeat sequence in viruses from Belarus, Ukraine, Lithuania and Poland [10]. This molecular marker is currently used in standard genotyping procedures. This additional repeat in reference to the Georgia 2007/1 strain was subsequently identified in all Central European strains, as well in Chinese domestic pigs, where also a second extra tandem repeat had been reported [11,12] Nevertheless, no additional tandem repeat was found in wild boar in China, suggesting its pan-Siberian origin [13,14].

According to the recently published ASFV whole genomic sequences originated from Poland, *K145R* and *MGF 505-5R* genes contain single nucleotide polymorphisms (SNPs) specific to all Polish isolates in comparison to the referenced Georgia 2007/1 strain [2]. The mutations are absent in the other viruses currently circulating in Europe and China, except the Ukraine/Kyiv/2016 strain [6]. This may suggest that these regions may become the potential molecular markers, which could provide tracking of the disease spreading.

The previous investigations showed that some ASFV isolates from Poland possess a unique tandem repeat of 14 nt within the *O174L* gene [9]. This insertion was identified specifically in the district of Biała Podlaska, Łosice and Siemiatycze during the period 2016–2017, and in the neighborhood of Warsaw between 2017 and 2018. ASFV epidemics nearby Warsaw city began at the end of 2017, and in 2019 over 1500 infected wild boars were confirmed within this cluster. Moreover, from the beginning of the disease epidemiology, two distinct clusters might be observed there, namely the southeastern and northwestern clusters to Warsaw, separated by the center of the city and S8 expressway [15]. The previous study showed that all tested samples, which were located in the southeastern cluster, possess a unique tandem repeat within the *O174L* gene [9]. For this reason, the *O174L* gene was selected for further investigation on the ASFV spread in Poland.

Sequence variations within the sequence of the *O174L* gene detected in this study showed that cases in eastern Poland are probably originating from the southeastern Warsaw cluster. Moreover, the northwestern Warsaw cluster does not possess SNPs specific to other Polish ASFVs, which may lead to a conclusion of its completely foreign, but still unknown origin. The *IGR* sequencing revealed a new, previously undescribed variant of the *IGR* region (IGR IV) with an additional three tandem repeats of 10 nt, which were found in isolates from wild boars and domestic pigs in the Warmińsko-Mazurskie voivodeship.

## 2. Materials and Methods

### 2.1. Sample Preparation to NGS Sequencing

#### 2.1.1. Cells and Viruses

In order to obtain high quality DNA to further the NGS sequencing, ASFV was isolated in primary porcine alveolar macrophages (PPAM) cell culture as previously described [2]. In total, the homogenates of 12 PCR-positive tissues (2 bone marrows, 6 spleens, 4 lungs) collected at the beginning of the epidemics in western Poland were subjected to virus isolation; nevertheless, only 7 produced the infective virus in the 2nd or the 3rd passage, which was confirmed by presence of hemadsorption phenomenon and real-time PCR. Out of them, one sample originated from bone marrow of the very first case confirmed in western Poland (Pol19_53050_C1959/19) was subjected to further NGS sequencing. Moreover, three additional ASFVs isolated from previous years, namely: Pol17_31177_O81 (Outbreak 81, Radzyń Podlaski province, Lubelskie voivodship, 2017), Pol17_55892_C754 (Case 754, Piaseczno province, Mazowieckie voivodship 2017), Pol18_28298_O111 (Outbreak 111, Chełm province, Lubelskie voivodship) were prepared and subjected to NGS sequencing as previously described [2]. Viral isolation and NGS sample preparation were performed under Biosafety level 3 (BSL3) conditions.

#### 2.1.2. DNA Extraction and Preparation to NGS Sequencing

The ASF virus Pol19_53050_C1959/19 was obtained on the 5th day of the 3rd passage in PPAM cell culture. Then, 2 × 200 µL of the cell culture was subjected to DNA extraction with a QiAMP DNA Mini Kit (Qiagen, Hilden, Germany). Final elution of DNA from the column was performed in triplicate (3 × 30 µL). Obtained DNA (2 × 90 µL) was pooled and cleaned-up using KAPA Hyper Pure Beads (Roche, Basel, Switzerland), and recovered in 20 µL of DNAse/RNAse free water. The final concentration of total DNA was measured with Nanodrop (Thermo Fisher Scientific, Waltham, MA, USA) (15.5 ng/µL), A260/A280 factor reached 1.86. The dsDNA concentration was measured using a Qbit dsDNA High Sensitivity kit (Thermo Fisher Scientific, Waltham, MA, USA) and reached 8.52 ng/µL. The remaining three isolates were prepared as described earlier [2].

#### 2.1.3. NGS Sequencing:

All samples were subjected to NGS sequencing using MiSeq instrument (Illumina, San Diego, CA, USA) in PE250 mode. NGS of two samples, specifically Pol18_28298_O111 and Pol19_53050_C1959/19, subsequent raw results analysis and consensus assembly was performed by an outsourcing company Genomed S.A. in Warsaw, Poland. Whole genome sequencing and consensus assembly of two remaining isolates, namely, Pol17_31177_O81 and Pol17_55892_C754, were performed thanks to the courtesy of colleagues from the Department of Omics Analyses at National Veterinary Research Institute (NVRI) in Puławy, Poland. Analysis of obtained results of any sequencing data was performed in Geneious R9 (BioMatters, Auckland, New Zealand), MEGA6 and MEGA X. The sequences have been submitted to GenBank database under the following accession numbers (in brackets): Pol17_31177_O81 (MT847622), Pol17_55892_C754 (MT847620), Pol18_28298_O111 (MT847621) and Pol19_53050_C1959/19 (MT847623).

### 2.2. Molecular Characterization by Sanger Sequencing

#### 2.2.1. Samples

In total, a number of 154 tissue samples collected from ASFV-positive animals collected between 2017 and 2020 were used for conventional sequencing of *O174L*, *K145R* and *IGR I73R/I329L*. Out of them, 72 samples were selected for sequencing of *MGF 505-5R* gene. Specimens were selected to cover all geographic regions where ASFV has been present up to now. The detailed characteristics of samples employed to this study, as well as obtained results for individual specimens, are presented in Supplementary Table S1. Total DNA was extracted from 200 µL 10% (*w*/*v*, PBS) tissue homogenates (bone marrow, spleen, lungs, lymph nodes, kidney, tonsil) or whole blood using QiaAmp DNA Mini kit (Qiagen, Hilden, Germany) or an automated extraction QiaCube system (Qiagen, Hilden, Germany), according to the manufacturer protocols. Tissue disruption was performed using TissueLyser II bead mill (Qiagen, Hilden, Germany). Extracted DNA was stored at −20 °C until further analysis. Viral genome was detected using a commercial Virotype (Indical Bioscience Gmbh, Leipzig, Germany) kit dedicated to ASFV diagnosis, including endogenous control of amplification. For further genotyping procedures, the samples that showed Cq < 30 were selected.

#### 2.2.2. Conventional PCR and Sequencing

Primers specific to selected regions were designed by Geneious R9. Detailed characteristics of the designed primers are included in Table 1.

Conventional PCR was carried using MyTaq HS Red Mix (Meridian Bioscience, Cincinnati, OH, USA), with a total volume of 25 µL, containing: 12.5 µL of Master Mix, 9.5 µL DNase RNase-free water, 1 µL of each primer in a final concentration of 40 pM, and 1 µL of DNA template. The following cycling protocol was applied: 1 cycle of 95 °C for 10 min, 35 cycles consisted of denaturation (95 °C for 15 s), annealing (50–52 °C for 30 s), and extension (72 °C for 30 s), followed by final extension at 72 °C for 3 min. The optimal annealing temperatures were set at 50 °C for *O174L*, *IGR*, and *K145R* and 52 °C for *MGF 505-5R* region. Amplification products were visualized by electrophoresis in 2.5% agarose gels (EurX, Gdańsk, Poland), stained by 20,000× diluted SimplySafe (EurX, Gdańsk, Poland) dye. Then, 100 bp DNA Ladder plus Gene Ruler (Thermo Fisher Scientific, Waltham, MA, USA) was used as the molecular weight marker. PCR products were subjected to conventional sequencing by the Sanger method, performed by an outsourcing service Genomed S.A. (Warsaw, Poland). Obtained raw nucleotide sequences were edited and analyzed using Geneious R9 (Biomatters, Auckland, New Zealand) software.

### 2.3. Phylogenetic Analysis

#### 2.3.1. Whole Genome Sequences

The resulting whole genome sequences were aligned to selected ones of closely related Eurasian genotype II isolates, with sequences available in the GenBank database, including reference Georgia 2007/1 (FR682468.1), Polish (MG939583-MG939589, MH681419), Ukrainian (MN194591), and others originated from China, Belgium, Hungary, Estonia, Lithuania, Moldova, Belgium and Czech Republic.

#### 2.3.2. Sequences Obtained by Conventional Sequencing of *O174L*, *K145R*, *MGF 505-5R* and *IGR I73R/I329L*

All gene sequences of Polish ASF viruses generated in this study were submitted to the GenBank database under the following accession numbers: MH764308.1, MH764311.1, MH764317.1– MH764319.1, MT304492–MT304607, MT951800–MT951827 for *O174L*; MT309188–MT309307, MT966756–MT966791 for *K145R*; MT889456–MT889527 for *MGF 505-5R* genes and MT889528–MT889625, MT951741–MT951797 for *IGR* region. In order to summarize and systematize the obtained results, phylogenetic analysis was performed for all isolates, based on the complete data on *O174L*, *K145R* and *IGR* sequences available. Due to full correlation between *K145R* and *MGF 505-5R*, the data on the latter were excluded. The concatenated sequences of selected genomic regions of investigated samples and the Georgia 2007/1 reference strain were aligned using CLUSTAL W package in Geneious R9 software. Subsequently, the unweighted pair group method with arithmetic mean (UPGMA) phylogenetic tree was constructed using the Jukes–Cantor distance model in Geneious R9 software; the obtained tree was then graphically edited in MEGA6 software.

### 2.4. Statistical Analysis

To evaluate correlations between occurrence of respective gene variants, a Pearson correlation analysis at a significance level 0.05 was performed using GraphPad Prism 8.4 (La Jolla, CA, USA) software.

### 2.5. Mapping

Maps of the geographical distribution of respective ASFV variants were prepared using MapHub beta online service, based on OpenStreetMap License. The analyzed samples were aggregated at the commune level. Locations within the commune were randomized.

## 3. Results

### 3.1. Next Generation Sequencing

The reads obtained during NGS sequencing were mapped to the reference, resulting in the consensus sequences of four analyzed isolates. The final assembled whole genome sequences varied from 189,405 to 189,422 bp in length. The nucleotide genomic alignment was performed to compare obtained sequences with other ASFV whole genome sequences belonging to genotype II; these are currently available at GenBank database. The conducted global alignment revealed from 99.678% to 99.992% similarity between analyzed sequences. All ASFV genomes generated in this study contained the variations typical to all previously described Polish isolates, namely: SNPs within *MGF 110-7L* (silent mutation), *MGF 505-5R* (Val->Ile) and *K145R* (Ser->Tyr) genes [2]. Moreover, two never described before point mutations (in reference to Georgia 2007/1 sequence) were detected: G63414A transition within the *K205R* gene (silent mutation) in Pol17_55892_C754 and Pol19_53050_C1959/19; C98939T transition within the *B475R* gene (Glu->Lys) exclusively in Pol18_28298_O111. As regards an additional tandem repeat within *O174L* gene, it was identified in Pol17_31177_O81, Pol17_55892_C754 and Pol19_53050_C1959/19, which was further confirmed by Sanger sequencing. Strain Pol18_28298_O111 does not contain this insertion.

### 3.2. Sanger Sequencing of Specific Regions

#### 3.2.1. *O174L* Gene

**Figure 1:**
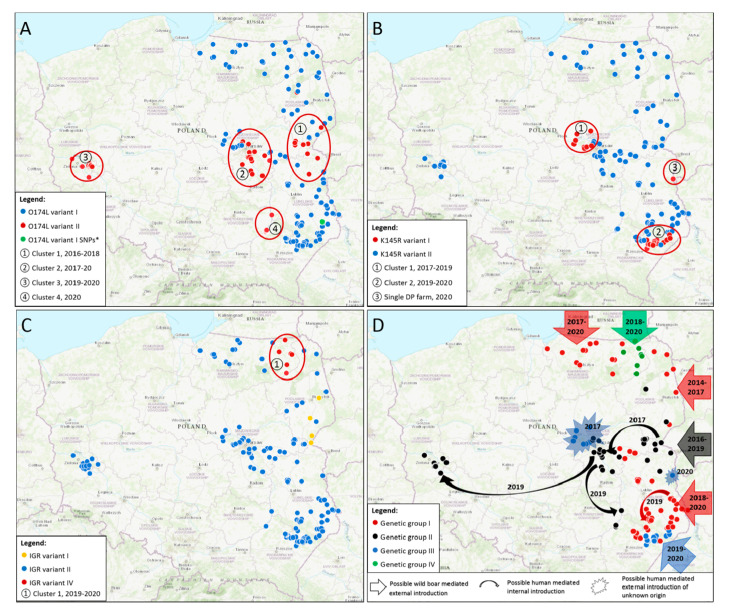
Spatial distribution of investigated gene variants of African swine fever virus (ASFV) in Poland during the period 2014–2020. Except for the data obtained during this study, the maps were supplemented with results from previous investigations [2,3,9] to improve the real picture of detected variations. (**A**)—*O174* gene. Blue dots ● represent variant I of the O174L gene, which shows 100% identity to the reference Georgia 2007/1 strain. Red dots ●represent variant II, containing a unique 14-nt insertion in this particular gene. *O174L* variant II formed four clusters (①②③④), separated in space and time. (**B**)—*K145R* gene. Red dots ● represent variant I of the *K145R* gene, which shows 100% identity to the reference Georgia 2007/1 strain and other published sequences from Eurasia (except Polish and Ukrainian ones). Blue dots ● represent variant II with a SNP specific to Poland [2] and confirmed also in a single Ukrainian African swine fever (ASF) genomic sequence [9]. *K145R* variant I formed two distinct clusters (①②), separated in space and time, moreover the single separated outbreak was confirmed in the eastern Poland (③). (**C**)—*IGR I73R/I329L* region. Yellow dots ● represent variant I of the IGR region, which shows 100% identity to the reference Georgia 2007/1. Blue dots ● represent variant II containing an insertion of 10 nt nucleotide sequence, representing a tandem repeat, which is the most abundant variant in European Union and Eastern Asia. Red dots ● represent IGR variant IV, identified for the first time in this study. IGR IV formed only one cluster, marked in eastern Warmińsko-Mazurskie voivodship. (**D**)—Genetic groups distinguished in Poland based on concatenated nucleotide data of *O174L*, *K145R* and *IGR I73R/I329L* and proposed virus introduction routes and transmission modes. Red dots ●represent genetic group I, black dots ●—group II, blue dots ●—group III, and green dots ●—group IV.

A number of 154 samples of ASF wild boar cases and outbreaks in pigs from 2017 to 2020 were used in this study. Maps were supplemented with data obtained in previous studies in order to improve the picture of ASFV epidemiology in time [2,9]. Most of the analyzed samples showed 100% sequence similarity to the Georgia 2007/1 strain, and other current genotype II strains from Europe and Asia, thus presenting variant I of the *O174L* gene. As expected, a 14-nt insertion of CAGTAGTGATTTTT presenting variant II of the *O174L* gene was observed in some samples, especially originated from the southern Warsaw cluster. In total, 50 out of 154 investigated samples (32.47%) possessed extra insertion within this region. At the regional resolution, in Mazowieckie the majority of investigated samples (21 out of 36; 58.33%) belonged to *O174L* variant II, as well as all samples in western Poland (14/14, 100%). The additional repeat was confirmed also in Podkarpackie (12/37, 32.43%), Podlaskie (1/5; 20%) and Lubelskie voivodeships (1/40; 2.5%); in Warmińsko-Mazurskie, all viruses belonged to O174L variant I. It has to be emphasized, that a marked distribution of mutation occurrence in space and time might be observed during the period 2014–2020 (Figure 1A, data from this study was supplemented with the previously obtained data [9]).

**Figure 2:**
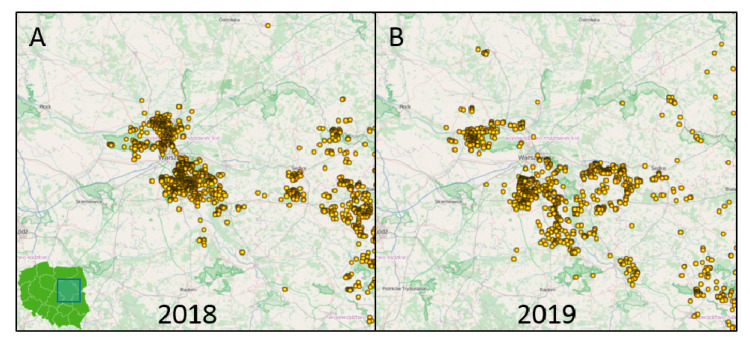
Spatial distribution of ASF cases in wild boars in the Warsaw cluster in: (**A**)—2018 and (**B**)—2019. Two geographically separated clusters are observed: northwestern and southeastern. The maps were derived from the website of the General Veterinary Inspectorate in Poland [16].

First cluster (Figure 1A, ①) of *O174L* variant II was observed between 2016 and 2018 in the eastern part of Mazowieckie, northern Lubelskie and southern Podlaskie, in the neighborhood of Belarussian border. The epizootic of second cluster (Figure 1A, ②), was related to the ASFV epidemics nearby Warsaw city which had begun unexpectedly at the end of 2017, about 100 km westward to the nearest confirmed cases in the eastern Mazowieckie. This specific region, called Warsaw cluster, presented as one of the most affected areas by then, therefore in 2018 and 2019 over 2600 ASF cases were confirmed there. Moreover, from the beginning of the disease epidemiology, two distinct clusters might be observed there: southeastern and northwestern to Warsaw, separated by the center of the city and expressway S8 (Figure 2) [15].

**Figure 3:**
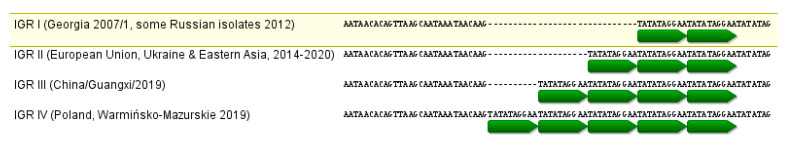
Partial nucleotide sequence alignment of the *IGR I73R/I329L* in various African swine fever virus (ASFV) isolates. The four known variants of this *IGR* are shown. Tandem repeat motifs are marked in green.

**Figure 4:**
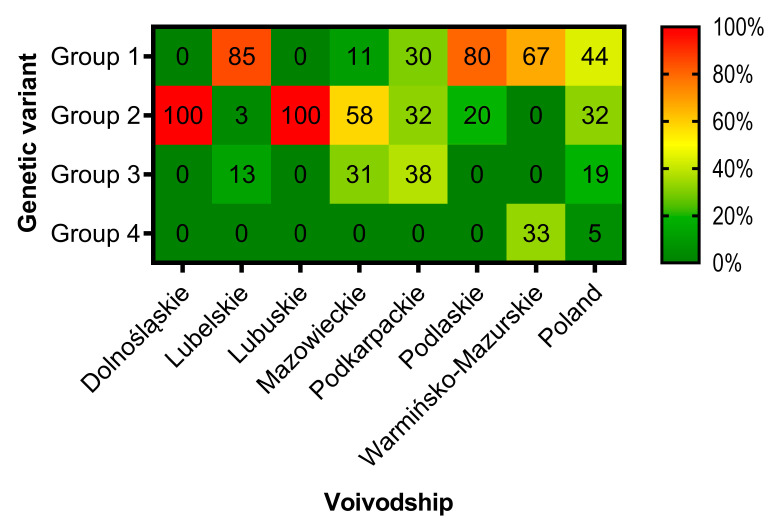
Frequency of determined ASFV genetic groups in Poland, 2017–2020. Each voivodship is presented separately.

Most samples originating from the southern Warsaw cluster possess an additional tandem repeat; detailed results are included in the Supplementary Table S1. As regards to recent epidemics in western Poland, all tested ASF isolates revealed the presence of the additional repeat, composing a third cluster (Figure 1A, ③) of *O174L* variant II. The last, fourth cluster (Figure 1A, ④), was observed in early 2020 in the Tarnobrzeg and Annopol districts, where the disease cases also emerged unexpectedly at a considerable distance of about 30 km from the nearest infected wild boars, and at an even greater distance of about 100 km from the nearest *O174L*-II cases in the southern Warsaw cluster. Occurrence of *O174L* variants showed a weak correlation with *K145R* variants (*R* = 0.37, *p* < 0.001). It should be emphasized that occurrence of O174L variant II excludes the K145R variant I (*R* = −1, *p* < 0.001), but O174L I may lead equally to K145R I or K145R II.

#### 3.2.2. *K145R* Gene

The conventional sequencing of the K145R gene was performed using the same sample panel as the *O174L* sequencing. The map in Figure 1B was supplemented with the data from NGS sequencing from this and the previous study [2]. The vast majority of investigated specimens (124 out of 154, 80.52%) showed the presence of SNP specific to Polish and Ukrainian isolates, e.g., C65167A transversion within *K145R* gene, in reference to Georgia 2007/1 strain nucleotide positions, representing *K145R* variant II (Figure 1B, blue dots). Unexpectedly, 30 out of 154 investigated samples (19.48%) revealed 100% similarity to reference Georgia 2007/1 strain. The first cluster (Figure 1B, ①) of the nucleotide pattern which is untypical in Poland, was related to disease emergence in the Warsaw cluster—specifically it was observed between 2017 and 2019 in the northern Warsaw cluster, with 11 confirmed cases of K145R variant I. The second cluster (Figure 1B, ②), comprised of the remaining 19 *K145R*-I, were located in southern Lubelskie and northern Podkarpackie voivodships, nearby Tomaszów Lubelski town. Interestingly, the epidemiology of ASFV in this particular region began from a single wild boar case in June 2019 (Pol19_29267_C1298/19, included in the study), located about 56 km from the nearest cases in the North and 22 km from the Ukrainian border. In total, 40 samples from this region were included in the analysis, but one of them originated from the 2018 epidemic, unrelated to the 2019 re-emergence. Nevertheless, the investigated samples from 2019 formed the cluster geographically separated from other cases, and these specific four cases belonged to *K145* variant I. The other minor cluster appeared also to the north of Tomaszów Lubelski; thus, similarly to the Warsaw cluster, nearby Tomaszów Lubelski there might be observed two sub-clusters: northern and southern ones. The southern sub-cluster continued to expand southwest, and the *K145R* variant was further confirmed in seven other wild boars and eight recent outbreaks in domestic pigs. These data suggest that ASFV emergence in Tomaszów Lubelski and neighboring counties in 2019 originated from abroad or the northern Warsaw cluster, highlighting a possible human activity factor in disease spreading. Nonetheless, considering the unknown *K145R* status for wild boars beyond the nearby Ukrainian border, the natural spreading of this specific virus genetic variant via wild boar migration could not be excluded. With regard to samples from the western Poland cluster, all of them belonged to *K145R* variant II, which is the most typical variant within the territory of Poland. Detailed results from this analysis are presented in Supplementary Table S1. Moreover, in mid-2020, *K145R*-I was confirmed in a single domestic pig farm located just at the border of Ukraine (Figure 1B, ③). Nevertheless, the epidemiological investigation did not reveal any possible sources of the virus; thus, the exact virus origin remains unknown.

#### 3.2.3. *IGR I73R/I329L* Region

The same panel of 154 samples was used to perform molecular characterization of this variable intergenic region. As expected, almost all investigated ASF isolates (147 out of 154, 95.45%) showed the presence of an additional tandem repeat of 10 nt, specific to the vast majority of current ASF genotype II viruses, including the European Union and eastern Asian strains (*IGR* variant II, Figure 1C, blue dots). Nevertheless, during the gel electrophoresis of obtained amplicons, the difference in size of some bands was observed, indicating the presence of products longer than expected. The sequencing revealed that these particular samples possess an additional two tandem repeats of 10 nt, in comparison to other Polish isolates, thus three tandems in comparison to Georgia 2007/1. This nucleotide pattern has never been described before, therefore, considering that *IGR* variant III contains one redundant tandem repeat in comparison to variant II, consequently we use the term of *IGR* variant IV for one more tandem repeat presence (Figure 3).

In total, 7 out of 154 investigated specimens (4.55%) have an untypical nucleotide pattern within this region. Analysis of their geographical origin showed that these cases were located exclusively in the eastern Warmińsko-Mazurskie voivodeship in the following districts: Olecko, Gołdap, Giżycko, Ełk. Epidemiological findings showed that the emergence of the disease in this voivodship began in the second half of 2017 in the western part of the region, just at the border of Kaliningrad Oblast. Nevertheless, the cases in the eastern part seemed to originate from distinct introduction, since the first cases there were distanced about 75 km from the western sub-cluster in Warmińsko-Mazurskie. The details of the obtained results are included in the Supplementary Table S1 and the geographical distribution of the investigated samples is presented in Figure 1C.

#### 3.2.4. *MGF 505-5R* Gene

*MGF 505-5R* gene sequencing was performed for 72 samples selected out of the investigated specimens. The vast majority of investigated specimens (57/72, 79.17%) showed the presence of SNP specific to Polish and Ukrainian isolates, thus G38332A transition within *MGF 505-5R* gene (*MGF 505-5R* variant II), in reference to Georgia 2007/1 strain nucleotide positions. Nevertheless, the 15 remaining samples (20.83%) revealed 100% similarity to reference Georgia 2007/1 strain (*MGF 505-5R* variant I), indicating their foreign origin. Interestingly, all of them belonged to the infrequent in Poland *K145R*-I. Pearson’s correlation coefficient was determined for the correlation between variant I of *K145R* and *MGF 505-5R*, showing full correlation between occurrence of specific variants in these two genes (*R* = 1, *p* < 0.001, *n* = 72).

### 3.3. Phylogenetic Analysis and Genetic Groups Differentiation

**Figure 5:**
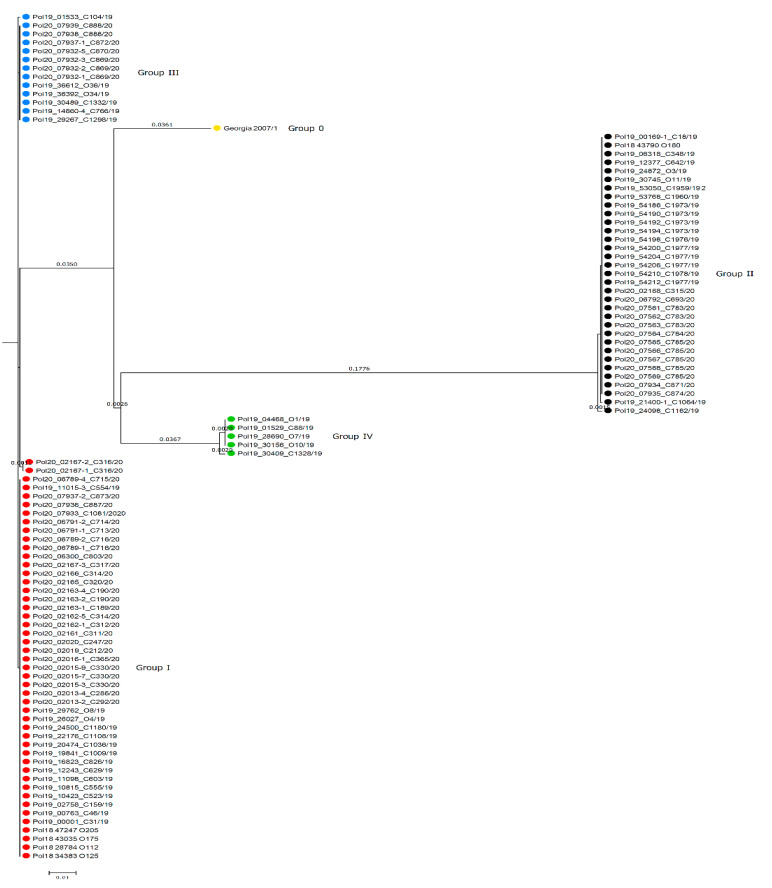
UPGMA phylogenetic tree constructed based on concatenated *O174L*, *K145R* and *IGR I73R/I329L* genomic regions sequences of ASFV present in Poland. Four main genetic groups (I–IV) are distinguished in Poland; the Georgia 2007/1 reference strain sequence formed a separate branch, representing group 0. Color of dot for each group corresponds to the group color on the map in Figure 1D. The scale bar indicates nucleotide substitutions per site.

Based on the concatenated nucleotide sequences of *O174L*, *K145R* and *IGR I73R/I329L* four genetic groups may be distinguished in Poland (Table 2, Figure 3, Figure 4 and Figure 5). The map presenting geographical distribution of individual groups is included in Figure 1D.

The overall nucleotide similarity between compared sequences varied from 96.355% to 100%. Group IV, comprised of seven isolates belonging to IGR IV, was the most divergent from other groups due to the highest number of nucleotide differences, ranging from 20 to 37 nt within an alignment of 1015 nt in length.

## 4. Discussion

Recombination and spontaneous mutation processes which occur during virus replication are the primary cause for virus genomic variability [17]. Similar to other dsDNA viruses, ASFV shows a moderate mutation rate, but is sufficient to differentiate its genome into 24 genotypes [18,19]. The investigation of virus molecular evolution in combination with spatio-temporal data is an integral part of pathogen tracing and may help in the identification of potential routes of its spreading, therefore in disease prevention and control [4,18,20]. Up to date, the molecular discrimination of various ASFV isolates was based mainly on the *B646L* gene and selected variable region sequencing [10,12,19,21,22]. The viruses responsible for current disease epizootic in Eurasia belonging to genotype II are routinely discriminated by *IGR I73R/I329L* sequencing [10], but recent study of Estonian isolates detected the variation in central variable region (*CVR*) within the *B602L* gene, which may support the geographical tracing of ASFVs [23]. Moreover, previously we confirmed a similar usefulness of *O174L* gene variation in such an investigation [9]. Another tandem repeat sequence variation located between *MGF 505-9R/10R* genes has also been described as a molecular marker [24]. Nowadays, wide use of NGS caused it to become more affordable, therefore the technique is commonly applied to ASFV genomic sequencing, especially in light of its considerable size of almost 200 kb [2,3,4,5,6,8,25,26,27]. Its usefulness for variation detection is invaluable; nonetheless, it is inadequate in terms of population studies.

In our study, the nucleotide variations detected within the whole genomic sequences of ASFV from Poland were evaluated as potential molecular markers, which may support tracing of disease spreading in space and time. Besides previously investigated *O174L* gene variation, the *IGR I73R/I329L* region, which is routinely used to perform molecular characteristics of worldwide distributed ASFV genotype II isolates, was employed. Furthermore, two SNPs: within *K145R* and *MGF 505-5R*, which, according to NGS results, were specific to Poland and Ukraine [2,6], were also evaluated as useful molecular tools for tracing disease spreading. Obtained results allowed for discrimination of four distinct genetic groups of ASFV circulating in Poland.

Group I (*O174L*-I, *K145R*-II, *IGR*-II) is the oldest, and the most common in the country, therefore it poses a “background” for the other groups.

Group II comprising the *O174L*-II variant, possessed an additional insertion of 14 nt, representing a tandem repeat. This group was initially identified in 2016 in close proximity to the Belarussian border, but subsequently it formed new, spatially distinct clusters: southwards to Warsaw (2017–2019), in western Poland (2019–2020), and at the border of Podkarpackie/Świętokrzyskie and Świętokrzyskie/Lubelskie voivodships (2020). Up to date, this variant is specific only to Poland and its exact origin is unknown, but considering disease epizootic and spreading, it may be hypothesized that it was introduced into Poland via natural migration of wild boars from Belarus in the period 2015–2016. Nevertheless, its further spreading was most likely associated with human activity, due to the fact that when this untypical variant occurred in the new location, it was always far-distanced from other confirmed cases or outbreaks, including the most spectacular jump into western Poland (Figure 1D, black arrows). All investigated samples from the western Poland cluster showed a homogenous nucleotide pattern, indicating their likely common origin, most probably due to single disease introduction into this area from the southern Warsaw cluster, which seriously suffered from ASF in wild boars directly preceding the timeframe (2017–2019). Similar, unexpected and geographically distinct disease introduction was observed also in Czech Republic in 2017 [28]. These incidents highlight that irresponsible and uncontrolled human activities are one of the main drivers of ASF transmission into disease-free regions, especially into wild-boar dense areas, where the disease spreads quickly, and can remain undetected for a long time.

Sequencing of *K145R* and *MGF 505-5R* revealed that most of the Polish ASFV isolates possess an SNP in comparison to Georgia 2007/1 and other Eurasian (except Ukrainian) strains. However, the Georgia-like gene version (called Group III, comprising *K145R-I/MGF 505-5R-I* isolates) was identified in characteristic locations in Mazowieckie (2017–2019), Podkarpackie and Lubelskie (2019–2020) voivodships. Statistical analysis showed full correlation between *K145R*-I and *MGF 505-5R*-I occurrence, indicating that these two variations might co-evolved together. In the case of Mazowieckie, *K145R*-I variant formed the separated northern Warsaw cluster that was discovered in the same timeframe as the southern one during intensified ASF surveillance in the region after detection of the first infected wild boar at the end of 2017. In the face of contemporary disease epizootics, it may be concluded that, similar to the western Poland cluster, the disease was most likely introduced there by humans. Moreover, with regard to the fact that this variant was then completely absent in Poland, it should be highlighted that the disease probably jumped into Mazowieckie from abroad, but its exact origin cannot be determined based on available molecular data. When it comes to the other uncommon *K145R* variants identified in Podkarpackie and Lubelskie, they compose a single cluster located in northeastern Podkarpackie and at the southern Lubelskie border. Molecular data in combination with the epidemiological one suggest that ASFV emergence here may have originated from abroad or the northern Warsaw cluster, again highlighting the possible human activity factor in disease spreading. However, with regard to the unknown *K145R* status for wild boars beyond the nearby Ukrainian border, the natural spreading of these specific virus variants via wild boar migration during the period 2019–2020 could not be excluded.

Sequencing of *IGR I73R/I329L* revealed that the vast majority (about 95%) of investigated samples show a homogenous nucleotide pattern in this region—being 100% identical to most of those identified in the current epidemic in Eurasia, which categorizes them into *IGR*-II. Nevertheless, we detected a brand new, never described before *IGR*-IV variant, possessing an additional three tandem repeats in comparison to the Georgia 2007/1 strain. This virus population also formed a homogenous and geographically distinct cluster in the northeastern Warmińsko-Mazurskie voivodship, having emerged in mid-2018 in the line of Gołdap–Olecko–Ełk cities. Most probably it originated from wild boars, which migrated from Kaliningrad Oblast. This discovery indicates that, despite the moderate usefulness of this region during current disease epidemics, especially after ASF introduction into EU, this region continues to evolve, therefore it still has great potential as a molecular marker.

In conclusion, the NGS sequencing of carefully selected ASFV isolates revealed numerous minor nucleotide variations in the virus genome. Nevertheless, these variations may become great molecular markers in terms of tracing the disease spreading. Our results confirmed that genomic regions containing tandem repeats could reveal disease trajectories in space and time. Due to technical issues, these regions are of particular interest in terms of standard genotyping procedures due to the difference in PCR product length, which is convenient to observe during regular agar electrophoresis. Moreover, we demonstrated that besides tandem repeats, SNPs, which are being identified during NGS sequencing, represent an attractive molecular tool that allows for discrimination of closely related ASFV strains. Based on the available whole genomic sequences deposited in the GenBank database, described variations up to date have not been detected in the majority of ASFVs currently circulating Eurasia (excluding Polish and Ukrainian strains); nevertheless, a wider population study might be required in order to definitively exclude their presence. Moreover, the introduction of a developed subtyping method into the routine diagnostic within affected worldwide areas, especially new disease excursions, may help in the identification of potential disease origins and provide a deeper understanding of spatio-temporal disease trajectories. Due to the low mutation rate of the ASFV genome and its slow molecular evolution, the usefulness of any subtyping within the same genotype is still limited and allows only for a moderate discrimination of closely related strains. Further investigation regarding other SNPs identified by whole genomic sequencing of isolates from other areas may help to determine potential disease trajectories at a higher resolution.

## Figures and Tables

**Table 1:** Characteristics of the primers used in the study. Amplification products of *O174L* covers the whole encoded gene, whereas *K145R* and *MGF 505-5R* primers were designed to cover only the regions containing the nucleotide of the interest.

Name	Sequence (5′->3′)	Position Referring to Georgia 2007/1 (FR682468.1)	Primer Length (nt)	Amplicon Length (nt)
O174L-F	TGGCTCAGACGATATTTCAACTC	128,160–128,182	23	672
O174L-R	GCCTCCACCACTTGAACCAT	128,832–128,813	20	
IGR-F	CTCAGAACTTTTTGAGAAGATTG	172,236–172,258	23	349
IGR-R	CAGCAAACAGTCCTATTGTT	172,585–172,566	20	
K145R-F	TTTCAGGCTGAAAACTTTTTAT	65,030–65,051	22	282
K145R-R	AAAGTTTTCAATGGTTGTTAGC	65,312–65,291	22	
MGF 505-5R-F	TACGCTTCTTTTCAATCATCAT	37,968–37,989	22	598
MGF 505-5R-R	AAATTAACAGTTGTTTGCCTTC	38,608–38,587	22	

**Table 2:** Genetic variants distinguished in Poland based on concatenated sequences of *K145R*, *O174L* and *IGR I73R/I329L*.

Genetic Group	*O174L* Variant	*K145R* Variant	*IGR* Variant	Occurrence (%)	Geographical Distribution
**0**	I	I	I	0	100% similarity to Georgia 2007/1, generally absent * in Poland.
**I**	I	II	II	43.51	The most typical for Poland.
**II**	II	II	II	32.47	Southern Warsaw, western Poland, Tarnobrzeg clusters.
**III**	I	I	II	19.48	Northern Warsaw, southern Tomaszów Lubelski clusters.
**IV**	I	II	IV	4.55	Eastern Warrmińsko-Mazurskie cluster.

* This variant has been detected several times by NGS sequencing [2,3], but it has not been confirmed by conventional sequencing.

## Supplementary Materials

The following are available online at https://www.mdpi.com/1999-4915/12/10/1094/s1, Table S1: Characteristics of samples used in the study.
