# Supplementary material for: The Spillover of African Swine Fever in Western Poland Revealed Its Estimated Origin on the Basis of O174L, K145R, MGF 505-5R and IGR I73R/I329L Genomic Sequences
Source: Viruses. 2020 Sep 27;12(10):1094. doi: 10.3390/v12101094 (PMC7601147; doi:10.3390/v12101094)
Supplement: Supplementary file 1 [file viruses-12-01094-s001.pdf]

Supplementary Table 1. Characteristics of samples used in the study.
*Case (C) – ASFV confirmed in wild boar or group of wild boars found in the same place, Outbreak (O) – ASFV confirmed at a single domestic pig farm.

#	Isolate name	Year	Case/ Outbreak no.	Voivodeship	District	County	*O174L*		*K145R*		*MGF 505-5R*		*IGR I73R/I329L*		Genetic group
							Gene variant	Accession number	Gene variant	Accession number	Gene variant	Accession number	Gene variant	Accession number	
1	**Pol17_53450_C675**	2017	C675	Mazowieckie	Warszawa	Kępa Zawadowska	II	MH764308.1	II	MT309188	II	MT889456	II	MT951741	2
2	**Pol17_55230_C728**	2017	C728	Mazowieckie	Piaseczno	Konstancin Jeziorna	II	MT304492	II	MT309189	II	MT889457	II	MT951742	2
3	**Pol17_55887_C756**	2017	C756	Mazowieckie	Piaseczno	Konstancin Jeziorna	II	MT304493	II	MT309190	II	MT889458	II	MT951743	2
4	**Pol17_56192_C799**	2017	C799	Mazowieckie	Siedlce	Przesmyki	II	MT304494	II	MT309191	II	MT889459	II	MT951744	2
5	**Pol17_56193_C800**	2017	C800	Mazowieckie	Siedlce	Korczew	II	MT304495	II	MT309192	II	MT889460	II	MT951745	2
6	**Pol17_56458_C792**	2017	C792	Mazowieckie	Piaseczno	Brześce	II	MT304496	II	MT309193	II	MT889461	II	MT951746	2
7	**Pol17_56772_C830**	2017	C830	Mazowieckie	Leszno	Zaborów	I	MT304497	I	MT309194	I	MT889462	II	MT951748	3
8	**Pol17_58258_C918**	2017	C918	Mazowieckie	Warszawa Zachodnia	Izabelin	I	MT304498	I	MT309195	I	MT889463	II	MT951749	3
9	**Pol17_58449-1_C921**	2017	C921	Mazowieckie	Siedlce	Korczew	II	MT304499	II	MT309196	II	MT889464	II	MT951750	2
10	**Pol17_58449-3_C921**	2017	C921	Mazowieckie	Siedlce	Korczew	II	MT304500	II	MT309197	II	MT889465	II	MT951751	2
11	**Pol17_58449-4_C921**	2017	C921	Mazowieckie	Siedlce	Korczew	II	MT304501	II	MT309198	II	MT889466	II	MT951752	2
12	**Pol17_58449-5_C921**	2017	C921	Mazowieckie	Siedlce	Korczew	II	MT304502	II	MT309199	II	MT889467	II	MT951753	2
13	**Pol17_58544_C923**	2017	C923	Mazowieckie	Piaseczno	Konstancin Jeziorna	II	MT304503	II	MT309200	II	MT889468	II	MT951754	2
14	**Pol17_58573_C932**	2017	C932	Mazowieckie	Warszawa Zachodnia	Leszno	I	MT304504	I	MT309201	I	MT889469	II	MT951755	3
15	**Pol18_00662_C958**	2018	C958	Mazowieckie	Wołomin	Marki	II	MT304505	II	MT309202	II	MT889470	II	MT951756	2
16	**Pol18_02942_C1145**	2018	C1145	Mazowieckie	Warszawa Zachodnia	Izabelin	I	MT304506	I	MT309203	I	MT889471	II	MT951757	3
17	**Pol18_02943_C1145**	2018	C1145	Mazowieckie	Warszawa Zachodnia	Izabelin	I	MT304507	I	MT309204	I	MT889472	II	MT951758	3
18	**Pol18_02944_C1145**	2018	C1145	Mazowieckie	Warszawa Zachodnia	Izabelin	I	MT304508	I	MT309205	I	MT889473	II	MT951759	3
19	**Pol18_07760_C1463**	2018	C1463	Mazowieckie	Warszawa Zachodnia	Łomianki	II	MT304509	II	MT309206	II	MT889474	II	MT951760	2
20	**Pol18_27608_O109**	2018	O109	Mazowieckie	Nowy Dwór Mazowiecki	Nasielsk	I	MT304510	I	MT309207	I	MT889475	II	MT951761	3
21	**Pol18_28784_O112**	2018	O112	Podlaskie	Sejny	Giby	I	MH764319.1	II	MT309208	II	MT889476	II	MT889528	1
22	**Pol18_34383_O125**	2018	O125	Podlaskie	Sokółka	Dąbrowa Białostocka	I	MH764318.1	II	MT309209	II	MT889477	II	MT889529	1
23	**Pol18_43035_O175**	2018	O175	Lubelskie	Lubartów	Ostrów Lubelski	I	MH764317.1	II	MT309210	II	MT889478	II	MT889530	1
24	**Pol18_43790_O180**	2018	O180	Mazowieckie	Mińsk Mazowiecki	Siennica	II	MH764311.1, MT304511	II	MT309211	II	MT889479	II	MT889531	2
25	**Pol18_47247_O205**	2018	O205	Podkarpackie	Lubaczów	Cieszanów	I	MT304512	II	MT309212	II	MT889480	II	MT889532	1

**26**	**Pol19_00001_C31/19**	2019	C31/2019	Lubelskie	Chełm	Kamień	I	MT304513	II	MT309213	II	MT889481	II	MT889533	1
**27**	**Pol19_00169-1_C18/19**	2019	C18/2019	Mazowieckie	Piaseczno	Lesznowola	II	MT304514	II	MT309214	II	MT889482	II	MT889534	2
**28**	**Pol19_00763_C46/19**	2019	C46/2019	Podlaskie	Suwałki	Suwałki	I	MT304515	II	MT309215	II	MT889483	II	MT889535	1
**29**	**Pol19_01529_C88/19**	2019	C88/2019	Warmińsko-Mazurskie	Olecko	Kowale Oleckie	I	MT304516	II	MT309216	II	MT889484	IV	MT889536	4
**30**	**Pol19_01533_C104/19**	2019	C104/2019	Mazowieckie	Warszawa Zachodnia	Leszno	I	MT304517	I	MT309217	I	MT889485	II	MT889537	3
**31**	**Pol19_02758_C159/19**	2019	C159/2019	Mazowieckie	Siedlce	Wiśniew	I	MT304518	II	MT309218	II	MT889486	II	MT889538	1
**32**	**Pol19_04468_O1/19**	2019	O1/2019	Warmińsko-Mazurskie	Gołdap	Gołdap	I	MT304519	II	MT309219	II	MT889487	IV	MT889539	4
**33**	**Pol19_06318_C348/19**	2019	C348/2019	Mazowieckie	Kozienice	Grabów nad Pilicą	II	MT304520	II	MT309220	II	MT889488	II	MT889540	2
**34**	**Pol19_10423_C523/19**	2019	C523/2019	Warmińsko-Mazurskie	Bartoszyce	Bartoszyce	I	MT304521	II	MT309221	II	MT889489	II	MT889541	1
**35**	**Pol19_10815_C555/19**	2019	C555/2019	Lubelskie	Chełm	Chełm	I	MT304522	II	MT309222	II	MT889490	II	MT889542	1
**36**	**Pol19_11015-3_C554/19**	2019	C554/2019	Warmińsko-Mazurskie	Bartoszyce	Bartoszyce	I	MT304523	II	MT309223	II	MT889491	II	MT889543	1
**37**	**Pol19_11098_C603/19**	2019	C603/2019	Mazowieckie	Mińsk Mazowiecki	Mrozy	I	MT304524	II	MT309224	II	MT889492	II	MT889544	1
**38**	**Pol19_12243_C629/19**	2019	C629/2019	Podlaskie	Sokółka	Szudziałowo	I	MT304525	II	MT309225	II	MT889493	II	MT889545	1
**39**	**Pol19_12377_C642/19**	2019	C642/2019	Mazowieckie	Grójec	Warka	II	MT304526	II	MT309226	II	MT889494	II	MT889546	2
**40**	**Pol19_14860-4_C766/19**	2019	C766/2019	Mazowieckie	Sochaczew	Brochów	I	MT304527	I	MT309227	I	MT889495	II	MT889547	3
**41**	**Pol19_16823_C826/19**	2019	C826/2019	Mazowieckie	Mińsk Mazowiecki	Mrozy	I	MT304528	II	MT309228	II	MT889496	II	MT889548	1
**42**	**Pol19_19841_C1009/19**	2019	C1009/2019	Warmińsko-Mazurskie	Lidzbark Warmiński	Lidzbark Warmiński	I	MT304529	II	MT309229	II	MT889497	II	MT889549	1
**43**	**Pol19_20474_C1036/19**	2019	C1036/2019	Mazowieckie	Siedlce	Skórzec	I	MT304530	II	MT309230	II	MT889498	II	MT889550	1
**44**	**Pol19_21400-1_C1064/19**	2019	C1064/2019	Mazowieckie	Nowy Dwór Mazowiecki	Jałonna	II	MT304531	II	MT309231	II	MT889499	II	MT889551	2
**45**	**Pol19_22176_C1108/19**	2019	C1108/2019	Lubelskie	Łuków	Stoczek Łukowski	I	MT304532	II	MT309232	II	MT889500	II	MT889552	1
**46**	**Pol19_24098_C1162/19**	2019	C1162/2019	Mazowieckie	Otwock	Wiązowna	II	MT304533	II	MT309233	II	MT889501	II	MT889553	2
**47**	**Pol19_24500_C1180/19**	2019	C1180/2019	Lubelskie	Chełm	Chełm	I	MT304534	II	MT309234	II	MT889502	II	MT889554	1
**48**	**Pol19_24872_O3/19**	2019	O3/2019	Podlaskie	Bielsk Podlaski	Orla	II	MT304535	II	MT309235	II	MT889503	II	MT889555	2
**49**	**Pol19_26027_O4/19**	2019	O4/2019	Lubelskie	Krasnystaw	Izbica	I	MT304536	II	MT309236	II	MT889504	II	MT889556	1
**50**	**Pol19_28690_O7/19**	2019	O7/2019	Warmińsko-Mazurskie	Giżycko	Kruklanki	I	MT304537	II	MT309237	II	MT889505	IV	MT889557	4
**51**	**Pol19_29267_C1298/19**	2019	C1298/19	Lubelskie	Tomaszów Lubelski	Tomaszów Lubelski	I	MT304538	I	MT309238	I	MT889506	II	MT889558	3
**52**	**Pol19_29762_O8/19**	2019	O8/2019	Warmińsko-Mazurskie	Węgorzewo	Budry	I	MT304539	II	MT309239	II	MT889507	II	MT889559	1
**53**	**Pol19_30156_O10/19**	2019	O10/2019	Warmińsko-Mazurskie	Olecko	Olecko	I	MT304540	II	MT309240	II	MT889508	IV	MT889560	4
**54**	**Pol19_30409_C1328/19**	2019	C1328/2019	Warmińsko-Mazurskie	Ełk	Ełk	I	MT304541	II	MT309241	II	MT889509	IV	MT889561	4

55	**Pol19_30489_C1332/19**	2019	C1332/2019	Mazowieckie	Płońsk	Naruszewo	I	MT304542	I	MT309242	I	MT889510	II	MT889562	3
56	**Pol19_30745_O11/19**	2019	O11/2019	Mazowieckie	Garwolin	Maciejowice	II	MT304543	II	MT309243	II	MT889511	II	MT889563	2
57	**Pol19_36392_O34/19**	2019	O34/2019	Lubelskie	Tomaszów Lubelski	Bełżec	I	MT304544	I	MT309244	I	MT889512	II	MT889564	3
58	**Pol19_36612_O36/19**	2019	O36/2019	Mazowieckie	Płońsk	Czerwińsk nad Wisłą	I	MT304545	I	MT309245	I	MT889513	II	MT889565	3
59	**Pol19_53050_C1959/19**	2019	C1959/2019	Lubuskie	Wschowa	Sława	II	MT304546	II	MT309246	II	MT889514	II	MT889566	2
60	**Pol19_53768_C1960/19**	2019	C1960/2019	Lubuskie	Wschowa	Nowa sól	II	MT304547	II	MT309247	II	MT889515	II	MT889567	2
61	**Pol19_54186_C1973/19**	2019	C1973/2019	Lubuskie	Wschowa	Nowa sól	II	MT304548	II	MT309248	II	MT889516	II	MT889568	2
62	**Pol19_54190_C1973/19**	2019	C1973/2019	Lubuskie	Wschowa	Nowa sól	II	MT304549	II	MT309249	II	MT889517	II	MT889569	2
63	**Pol19_54192_C1973/19**	2019	C1973/2019	Lubuskie	Wschowa	Nowa sól	II	MT304550	II	MT309250	II	MT889518	II	MT889570	2
64	**Pol19_54194_C1973/19**	2019	C1973/2019	Lubuskie	Wschowa	Nowa sól	II	MT304551	II	MT309251	II	MT889519	II	MT889571	2
65	**Pol19_54198_C1976/19**	2019	C1976/2019	Lubuskie	Wschowa	Nowa sól	II	MT304552	II	MT309252	II	MT889520	II	MT889572	2
66	**Pol19_54200_C1977/19**	2019	C1977/2019	Lubuskie	Wschowa	Nowa sól	II	MT304553	II	MT309253	II	MT889521	II	MT889573	2
67	**Pol19_54204_C1977/19**	2019	C1977/2019	Lubuskie	Wschowa	Nowa sól	II	MT304554	II	MT309254	II	MT889522	II	MT889574	2
68	**Pol19_54206_C1977/19**	2019	C1977/2019	Lubuskie	Wschowa	Nowa sól	II	MT304555	II	MT309255	II	MT889523	II	MT889575	2
69	**Pol19_54210_C1978/19**	2019	C1978/2019	Lubuskie	Wschowa	Kolsko	II	MT304556	II	MT309256	II	MT889524	II	MT889576	2
70	**Pol19_54212_C1978/19**	2019	C1978/2019	Lubuskie	Wschowa	Kolsko	II	MT304557	II	MT309257	II	MT889525	II	MT889577	2
71	**Pol19_55190-6_C2032/19**	2019	C2032/2019	Podkarpackie	Lubaczów	Narol	I	MT304558	I	MT309258	I	MT889526	II	MT951762	3
72	**Pol19_55195-1_C2058/19**	2019	C2058/2019	Podkarpackie	Lubaczów	Cieszanów	I	MT304559	I	MT309259	I	MT889527	II	MT951763	3
73	**Pol20_02013-2_C292/20**	2020	C292/2020	Warmińsko-Mazurskie	Giżycko	Ryn	I	MT304560	II	MT309260	n/a	n/a	II	MT889578	1
74	**Pol20_02013-4_C286/20**	2020	C286/2020	Warmińsko-Mazurskie	Giżycko	Ryn	I	MT304561	II	MT309261	n/a	n/a	II	MT889579	1
75	**Pol20_02015-3_C330/20**	2020	C330/2020	Warmińsko-Mazurskie	Elbląg	Godkowo	I	MT304562	II	MT309262	n/a	n/a	II	MT889580	1
76	**Pol20_02015-7_C330/20**	2020	C330/2020	Warmińsko-Mazurskie	Elbląg	Godkowo	I	MT304563	II	MT309263	n/a	n/a	II	MT889581	1
77	**Pol20_02015-9_C330/20**	2020	C330/2020	Warmińsko-Mazurskie	Elbląg	Godkowo	I	MT304564	II	MT309264	n/a	n/a	II	MT889582	1
78	**Pol20_02016-1_C365/20**	2020	C365/2020	Warmińsko-Mazurskie	Elbląg	Elbląg	I	MT304565	II	MT309265	n/a	n/a	II	MT889583	1
79	**Pol20_02019_C212/20**	2020	C212/2020	Warmińsko-Mazurskie	Olsztyn	Dywity	I	MT304566	II	MT309266	n/a	n/a	II	MT889584	1
80	**Pol20_02020_C247/20**	2020	C247/2020	Warmińsko-Mazurskie	Pisz	Ruciane-Nida	I	MT304567	II	MT309267	n/a	n/a	II	MT889585	1
81	**Pol20_02161_C311/20**	2020	C311/2020	Lubelskie	Biłgoraj	Turobin	I	MT304568	II	MT309268	n/a	n/a	II	MT889624	1
82	**Pol20_02162-1_C312/20**	2020	C312/2020	Lubelskie	Tomaszów Lubelski	Rachanie	I	MT304569	II	MT309269	n/a	n/a	II	MT889586	1
83	**Pol20_02162-5_C314/20**	2020	C314/2020	Lubelskie	Tomaszów Lubelski	Tarnawatka	I	MT304570	II	MT309270	n/a	n/a	II	MT889587	1
84	**Pol20_02163-1_C189/20**	2020	C189/2020	Podkarpackie	Nisko	Rudnik nad Sanem	I	MT304571	II	MT309271	n/a	n/a	II	MT889588	1

**85**	**Pol20_02163-2_C190/20**	2020	C190/2020	Podkarpackie	Nisko	Rudnik nad Sanem	I	MT304572	II	MT309272	n/a	n/a	II	MT889589	1
**86**	**Pol20_02163-4_C190/20**	2020	C190/2020	Podkarpackie	Nisko	Rudnik nad Sanem	I	MT304573	II	MT309273	n/a	n/a	II	MT889590	1
**87**	**Pol20_02165_C320/20**	2020	C320/2020	Lubelskie	Tomaszów Lubelski	Rachanie	I	MT304574	II	MT309274	n/a	n/a	II	MT889591	1
**88**	**Pol20_02166_C314/20**	2020	C314/2020	Lubelskie	Tomaszów Lubelski	Tarnawatka	I	MT304575	II	MT309275	n/a	n/a	II	MT889592	1
**89**	**Pol20_02167-1_C316/20**	2020	C316/2020	Lubelskie	Zamość	Miączyn	I	MT304576	II	MT309276	n/a	n/a	II	MT889593	1
**90**	**Pol20_02167-2_C316/20**	2020	C316/2020	Lubelskie	Zamość	Miączyn	I	MT304577	II	MT309277	n/a	n/a	II	MT889594	1
**91**	**Pol20_02167-3_C317/20**	2020	C317/2020	Lubelskie	Zamość	Komarów Osada	I	MT304578	II	MT309278	n/a	n/a	II	MT889595	1
**92**	**Pol20_02168_C315/20**	2020	C315/2020	Lubelskie	Kraśnik	Annopol	II	MT304579	II	MT309279	n/a	n/a	II	MT889596	2
**93**	**Pol20_06300_C803/20**	2020	C803/2020	Lubelskie	Lublin	Wysokie	I	MT304580	II	MT309280	n/a	n/a	II	MT889597	1
**94**	**Pol20_06789-1_C716/20**	2020	C716/2020	Podkarpackie	Nisko	Rudnik nad Sanem	I	MT304581	II	MT309281	n/a	n/a	II	MT889598	1
**95**	**Pol20_06789-2_C716/20**	2020	C716/2020	Podkarpackie	Nisko	Rudnik nad Sanem	I	MT304582	II	MT309282	n/a	n/a	II	MT889599	1
**96**	**Pol20_06789-4_C715/20**	2020	C715/2020	Podkarpackie	Nisko	Rudnik nad Sanem	I	MT304583	II	MT309283	n/a	n/a	II	MT889625	1
**97**	**Pol20_06791-1_C713/20**	2020	C713/2020	Podkarpackie	Leżajsk	Kuryłówka	I	MT304584	II	MT309284	n/a	n/a	II	MT889600	1
**98**	**Pol20_06791-2_C714/20**	2020	C714/2020	Podkarpackie	Leżajsk	Kuryłówka	I	MT304585	II	MT309285	n/a	n/a	II	MT889601	1
**99**	**Pol20_06792_C693/20**	2020	C693/2020	Podkarpackie	Tarnobrzeg	Tarnobrzeg	II	MT304586	II	MT309286	n/a	n/a	II	MT889602	2
**100**	**Pol20_07561_C783/20**	2020	C783/2020	Podkarpackie	Tarnobrzeg	Tarnobrzeg	II	MT304587	II	MT309287	n/a	n/a	II	MT889603	2
**101**	**Pol20_07562_C783/20**	2020	C783/2020	Podkarpackie	Tarnobrzeg	Tarnobrzeg	II	MT304588	II	MT309288	n/a	n/a	II	MT889604	2
**102**	**Pol20_07563_C783/20**	2020	C783/2020	Podkarpackie	Tarnobrzeg	Tarnobrzeg	II	MT304589	II	MT309289	n/a	n/a	II	MT889605	2
**103**	**Pol20_07564_C784/20**	2020	C784/2020	Podkarpackie	Tarnobrzeg	Tarnobrzeg	II	MT304590	II	MT309290	n/a	n/a	II	MT889606	2
**104**	**Pol20_07565_C785/20**	2020	C785/2020	Podkarpackie	Tarnobrzeg	Tarnobrzeg	II	MT304591	II	MT309291	n/a	n/a	II	MT889607	2
**105**	**Pol20_07566_C785/20**	2020	C785/2020	Podkarpackie	Tarnobrzeg	Tarnobrzeg	II	MT304592	II	MT309292	n/a	n/a	II	MT889608	2
**106**	**Pol20_07567_C785/20**	2020	C785/2020	Podkarpackie	Tarnobrzeg	Tarnobrzeg	II	MT304593	II	MT309293	n/a	n/a	II	MT889609	2
**107**	**Pol20_07568_C785/20**	2020	C785/2020	Podkarpackie	Tarnobrzeg	Tarnobrzeg	II	MT304594	II	MT309294	n/a	n/a	II	MT889610	2
**108**	**Pol20_07569_C785/20**	2020	C785/2020	Podkarpackie	Tarnobrzeg	Tarnobrzeg	II	MT304595	II	MT309295	n/a	n/a	II	MT889611	2
**109**	**Pol20_07932-1_C869/20**	2020	C869/2020	Podkarpackie	Lubaczów	Stary Dzików	I	MT304596	I	MT309296	n/a	n/a	II	MT889612	3
**110**	**Pol20_07932-2_C869/20**	2020	C869/2020	Podkarpackie	Lubaczów	Stary Dzików	I	MT304597	I	MT309297	n/a	n/a	II	MT889613	3
**111**	**Pol20_07932-3_C869/20**	2020	C869/2020	Podkarpackie	Lubaczów	Stary Dzików	I	MT304598	I	MT309298	n/a	n/a	II	MT889614	3
**112**	**Pol20_07932-5_C870/20**	2020	C870/2020	Podkarpackie	Lubaczów	Stary Dzików	I	MT304599	I	MT309299	n/a	n/a	II	MT889615	3
**113**	**Pol20_07933_C1081/20**	2020	C1081/2020	Lubelskie	Hrubieszów	Uchanie	I	MT304600	II	MT309300	n/a	n/a	II	MT889616	1
**114**	**Pol20_07934_C871/20**	2020	C871/2020	Podkarpackie	Tarnobrzeg	Tarnobrzeg	II	MT304601	II	MT309301	n/a	n/a	II	MT889617	2
**115**	**Pol20_07935_C874/20**	2020	C874/2020	Podkarpackie	Tarnobrzeg	Tarnobrzeg	II	MT304602	II	MT309302	n/a	n/a	II	MT889618	2
**116**	**Pol20_07936_C887/20**	2020	C887/2020	Podkarpackie	Nisko	Krzeszów	I	MT304603	II	MT309303	n/a	n/a	II	MT889619	1

**117**	**Pol20_07937-1_C873/20**	2020	C873/2020	Podkarpackie	Leżajsk	Kuryłówka	I	MT304604	I	MT309304	n/a	n/a	II	MT889620	3
**118**	**Pol20_07937-2_C872/20**	2020	C872/2020	Podkarpackie	Leżajsk	Sarzyna	I	MT304605	II	MT309305	n/a	n/a	II	MT889621	1
**119**	**Pol20_07938_C888/20**	2020	C888/2020	Podkarpackie	Przeworsk	Adamówka	I	MT304606	I	MT309306	n/a	n/a	II	MT889622	3
**120**	**Pol20_07939_C888/20**	2020	C888/2020	Podkarpackie	Przeworsk	Adamówka	I	MT304607	I	MT309307	n/a	n/a	II	MT889623	3
**121**	**Pol20_12821-24_O1/20**	2020	O1/2020	Lubuskie	Nowa sól	Otyń	II	MT951800	II	MT966756	n/a	n/a	II	MT951764	2
**122**	**Pol20_24362-3_O3/20**	2020	O3/2020	Lubelskie	Włodawa	Włodawa	I	MT951820	I	MT966757	n/a	n/a	II	MT951765	3
**123**	**Pol20_27023_O5/20**	2020	O5/2020	Dolnośląskie	Polkowice	Gaworzyce	II	MT951802	II	MT966758	n/a	n/a	II	MT951766	2
**124**	**Pol20_29419-3_O6/20**	2020	O6/2020	Warmińsko-Mazurskie	Olecko	Olecko	I	MT951828	II	MT966759	n/a	n/a	IV	MT951767	4
**125**	**Pol20_31597_O7/20**	2020	O7/2020	Lubelskie	Biłgoraj	Obsza	I	MT951829	I	MT966760	n/a	n/a	II	MT951768	3
**126**	**Pol20_32301-5_O8/20**	2020	O8/2020	Lubelskie	Zamość	Radecznica	I	MT951830	II	MT966761	n/a	n/a	II	MT951769	1
**127**	**Pol20_32500_O9/20**	2020	O9/2020	Warmińsko-Mazurskie	Lidzbark Warmiński	Lidzbark Warmiński	I	MT951831	II	MT966762	n/a	n/a	II	MT951770	1
**128**	**Pol20_32607_O11/20**	2020	O11/2020	Podkarpackie	Lubaczów	Stary Dzików	I	MT951832	I	MT966763	n/a	n/a	II	MT951771	3
**129**	**Pol20_32662_O12/20**	2020	O12/2020	Lubelskie	Biłgoraj	Frampol	I	MT951833	II	MT966764	n/a	n/a	II	MT951772	1
**130**	**Pol20_32957_O14/20**	2020	O14/2020	Lubelskie	Biłgoraj	Frampol	I	MT951803	II	MT966765	n/a	n/a	II	MT951773	1
**131**	**Pol20_32983_O15/20**	2020	O15/2020	Warmińsko-Mazurskie	Ełk	Prostki	I	MT951804	II	MT966766	n/a	n/a	IV	MT951774	4
**132**	**Pol20_33449_O17/20**	2020	O17/2020	Lubuskie	Zielona góra	Czerwieńsk	II	MT951798	II	MT966767	n/a	n/a	II	MT951775	2
**133**	**Pol20_33591_O19/20**	2020	O19/2020	Warmińsko-Mazurskie	Olsztyn	Dywity	I	MT951805	II	MT966768	n/a	n/a	II	MT951776	1
**134**	**Pol20_34099_O27/20**	2020	O27/2020	Lubelskie	Biłgoraj	Biłgoraj	I	MT951807	II	MT966769	n/a	n/a	II	MT951777	1
**135**	**Pol20_34102_O26/20**	2020	O26/2020	Lubelskie	Zamość	Radecznica	I	MT951808	II	MT966770	n/a	n/a	II	MT951778	1
**136**	**Pol20_34126_O31/20**	2020	O31/2020	Lubelskie	Lubartów	Michów	I	MT951809	II	MT966771	n/a	n/a	II	MT951779	1
**137**	**Pol20_34172_O29/20**	2020	O29/2020	Lubelskie	Chełm	Żmudź	I	MT951810	II	MT966772	n/a	n/a	II	MT951780	1
**138**	**Pol20_34185_O30/20**	2020	O30/2020	Mazowieckie	Garwolin	Trojanów	II	MT951801	II	MT966773	n/a	n/a	II	MT951781	2
**139**	**Pol20_34190-10_O32/20**	2020	O32/2020	Lubelskie	Biłgoraj	Goraj	I	MT951811	II	MT966774	n/a	n/a	II	MT951782	1
**140**	**Pol20_34410-2_O22/20**	2020	O22/2020	Lubelskie	Hrubieszów	Hrubieszów	I	MT951806	II	MT966775	n/a	n/a	II	MT951783	1
**141**	**Pol20_34480_O34/20**	2020	O34/2020	Lubelskie	Hrubieszów	Werbkowice	I	MT951812	II	MT966777	n/a	n/a	II	MT951784	1
**142**	**Pol20_34554_O42/20**	2020	O42/2020	Lubelskie	Lubartów	Michów	I	MT951816	II	MT966778	n/a	n/a	II	MT951785	1
**143**	**Pol20_34630_O38/20**	2020	O38/2020	Podkarpackie	Przeworsk	Adamówka	I	MT951814	I	MT966779	n/a	n/a	II	MT951786	3
**144**	**Pol20_34634_O37/20**	2020	O37/2020	Podkarpackie	Przeworsk	Adamówka	I	MT951813	I	MT966780	n/a	n/a	II	MT951787	3
**145**	**Pol20_35032_O45/20**	2020	O45/2020	Lubelskie	Hrubieszów	Horodło	I	MT951819	II	MT966781	n/a	n/a	II	MT951788	1
**146**	**Pol20_35056-5_O44/20**	2020	O44/2020	Lubelskie	Lubartów	Michów	I	MT951818	II	MT966782	n/a	n/a	II	MT951789	1
**147**	**Pol20_35150_O46/20**	2020	O46/2020	Lubelskie	Tomaszów Lubelski	Tarnawatka	I	MT951821	II	MT966784	n/a	n/a	II	MT951790	1
**148**	**Pol20_35226_O43/20**	2020	O43/2020	Lubelskie	Lubartów	Michów	I	MT951817	II	MT966785	n/a	n/a	II	MT951791	1

**149**	**Pol20_35277_O48/20**	2020	O48/2020	Lubelskie	Zamość	Radecznica	I	MT951823	II	MT966786	n/a	n/a	II	MT951792	1
**150**	**Pol20_35280-1_O40/20**	2020	O40/2020	Lubelskie	Hrubieszów	Hrubieszów	I	MT951815	II	MT966787	n/a	n/a	II	MT951793	1
**151**	**Pol20_35466_O50/20**	2020	O50/2020	Lubelskie	Biłgoraj	Obsza	I	MT951825	I	MT966788	n/a	n/a	II	MT951794	3
**152**	**Pol20_35644-3_O49/20**	2020	O49/2020	Lubelskie	Biłgoraj	Księżpol	I	MT951824	II	MT966789	n/a	n/a	II	MT951795	1
**153**	**Pol20_36116_O51/20**	2020	O51/2020	Podkarpackie	Przeworsk	Sieniawa	I	MT951826	I	MT966790	n/a	n/a	II	MT951796	3
**154**	**Pol20_36120_O52/20**	2020	O52/2020	Podkarpackie	Przeworsk	Sieniawa	I	MT951827	I	MT966791	n/a	n/a	II	MT951797	3
